# Obesity-Related Inflammation Reduces Treatment Sensitivity and Promotes Aggressiveness in Luminal Breast Cancer Modulating Oxidative Stress and Mitochondria

**DOI:** 10.3390/biomedicines12122813

**Published:** 2024-12-11

**Authors:** Pere Miquel Morla-Barcelo, Lucas Melguizo-Salom, Pilar Roca, Mercedes Nadal-Serrano, Jorge Sastre-Serra, Margalida Torrens-Mas

**Affiliations:** 1 Grupo Multidisciplinar de Oncología Traslacional, Institut Universitari d’Investigació en Ciències de la Salut (IUNICS), Universitat de les Illes Balears, 07122 Palma, Islas Baleares, Spain; pere.morla@uib.es (P.M.M.-B.); lucas.melguizo@uib.es (L.M.-S.); pilar.roca@uib.es (P.R.); mercedes.nadal@uib.es (M.N.-S.); lida.torrens@uib.es (M.T.-M.); 2 Institut d’Investigació Sanitària de les Illes Balears (IdISBa), 07120 Palma, Islas Baleares, Spain; 3 CIBER Fisiopatología Obesidad y Nutrición (CB06/03), Instituto de Salud Carlos III, 28029 Madrid, Spain

**Keywords:** obesity, luminal breast cancer, mammospheres, drug sensitivity, oxidative stress, mitochondria

## Abstract

Background: Obesity, characterized by the secretion of several pro-inflammatory cytokines and hormones, significantly increases the risk of developing breast cancer and is associated with poorer outcomes. Mitochondrial and antioxidant status are crucial in both tumor progression and treatment response. Methods: This study investigates the impact of an ELIT cocktail (17β-estradiol, leptin, IL-6, and TNFα), which simulates the obesity-related inflammation condition in postmenopausal women, using a 3D culture model. We examined the effects of ELIT exposure on mammosphere formation, oxidative stress and mitochondrial markers, and treatment sensitivity in luminal (T47D, MCF7) and triple-negative (MDA-MB-231) breast cancer cell lines. After that, 3D-derived cells were re-cultured under adherent conditions focusing on the mechanisms leading to dissemination and drug sensitivity. Results: Our results indicated that ELIT condition significantly increased mammosphere formation in luminal breast cancer cell lines (from 3.26% to 6.38% in T47D cell line and 0.68% to 2.32% in MCF7 cell line) but not in the triple-negative MDA-MB-231 cell line. Further analyses revealed a significant decrease in mitochondrial and antioxidant-related markers, particularly in the T47D cell line, where higher levels of *ESR2*, three-fold increased by ELIT exposure, may play a critical role. Importantly, 3D-derived T47D cells exposed to ELIT showed reduced sensitivity to tamoxifen and paclitaxel, avoiding a 34.2% and 75.1% reduction in viability, respectively. Finally, through in silico studies, we identified specific biomarkers, including *TOMM20*, *NFE2L2*, *CAT*, and *ESR2*, correlated with poor prognosis in luminal breast cancer. Conclusions: Taken together, our findings suggest that antioxidant and mitochondrial markers are key factors that reduce treatment sensitivity in obesity-related luminal breast cancer. The identified biomarkers may serve as valuable tools for the prognosis and development of more effective therapies in these patients.

## 1. Introduction

Obesity, a chronic metabolic disorder with an increasing prevalence in developed countries, is now widely recognized as a significant risk factor for postmenopausal breast cancer, particularly for luminal breast cancers [1,2]. It is estimated that obesity contributes to 4–8% of all cancer cases, with excess body fat associated with roughly a 17% higher risk of cancer-related mortality [3]. Additionally, data from pooled cohort studies reveal that women with central obesity have a 39% increased likelihood of developing breast cancer compared to those without central obesity [4]. Luminal breast cancer (estrogen receptor-positive) is the most prevalent breast cancer subtype, accounting for about 70% of all cases [5]. While triple-negative breast cancer (lacking estrogen, progesterone, and human epidermal growth factor receptor 2 expression) is considered the clinical subtype with the worst prognosis, the link between obesity and breast cancer is more associated with luminal subtypes. This association is largely attributed to elevated levels of circulating estrogens, particularly 17β-estradiol, which is produced by aromatase in adipose tissue [6,7]. 17β-estradiol binds to different estrogen receptor isoforms, alpha (ERα) and beta (ERβ), triggering various cellular responses, including cell proliferation and invasiveness [7,8]. Additionally, recent findings have highlighted the role of ERβ as a key player in obesity-related inflammation [7,9]. Obesity is also associated with the secretion of pro-inflammatory cytokines, such as interleukin-6 (IL-6) and tumor necrosis factor alpha (TNFα), as well as adipokines like leptin. Together, these signals create a tumor-promoting environment that enhances breast cancer initiation, progression, and metastasis [10,11,12].

It is well established that breast cancer patients with obesity tend to have a worse prognosis and reduced response to treatments [13,14]. This correlation has been attributed to various factors, including altered drug metabolism, increased inflammation, and changes in the tumor microenvironment driven by obesity [15,16]. Moreover, recent studies have suggested the role of obesity in promoting and accelerating the metastatic process in breast cancer, potentially through mechanisms involving chronic inflammation, dysregulated adipokine signaling, and enhanced tumor cell invasiveness [17,18]. These findings highlight the urgency of identifying novel therapeutic strategies to improve treatment outcomes for these patients.

Mitochondrial dysfunction related to obesity has emerged as a critical factor contributing to both drug sensitivity and metastatic progression in breast cancer [19]. This dysfunction leads to an imbalance between oxidative stress and antioxidant defenses, significantly impacting tumor cell survival; resistance to apoptosis; and, consequently, decreasing drug sensitivity [20,21]. Oxidative stress is not only involved in drug response but also in facilitating metastatic progression [22,23]. Dynamic levels of reactive oxygen species (ROS) in tumor cells, regulated by antioxidants, have been implicated in modulating different phases of the metastatic process, including intravasation, circulation, extravasation, and colonization [23]. For this reason, a deeper understanding of how mitochondrial oxidative stress and antioxidant systems are altered in the context of obesity and breast cancer is essential for developing more precise therapeutic approaches. Targeting oxidative stress-related mechanisms may provide new strategies to overcome drug resistance and prevent metastatic spread, ultimately improving treatment outcomes in breast cancer patients with obesity.

In this context, mammospheres—three-dimensional (3D) spherical clusters of breast cancer cells—are considered an in vitro model that better mimics the complex architecture of a solid tumor compared to traditional two-dimensional (2D) cell cultures [24]. Consequently, these 3D cultures are emerging as an excellent tool for studying the mechanisms of cancer progression and drug response in breast cancer [25,26,27].

Taking this together, the present study explores how mitochondrial oxidative stress and antioxidant systems are altered in the context of obesity and breast cancer, with the aim of identifying mechanisms involved in drug response and metastatic progression using a 3D culture model. These findings could ultimately lead to improved clinical outcomes for luminal breast cancer patients with obesity.

## 2. Materials and Methods

### 2.1. Materials

17β-estradiol, leptin, IL-6, TNF-α, Tamoxifen, and Paclitaxel were obtained from Merck (St. Louis, MO, USA). Human breast cancer cell lines T47D and MCF7 (luminal) and MDA-MB-231 (triple-negative) were obtained from American Type Culture Collection ATCC (Manassas, VA, USA). Biowest (Riverside, MO, USA) supplied Dulbecco’s modified Eagle’s medium (DMEM) High Glucose, pH 7.20 ± 0.30, while GIBCO (Paisley, UK) supplied DMEM without phenol red, pH 7.20 ± 0.20. Capricorn Scientific (Ebsdorfergrund, Germany) supplied Dulbecco’s Phosphate-buffered Saline (PBS), pH 7.25 ± 0.25, and Biological Industries (Kibbutz Beit Haemek, Israel) supplied Fetal Bovine Serum (FBS) and antibiotics (streptomycin and penicillin). Three-dimensional Tumorsphere Medium XF (3DTM) without phenol red was acquired from Promocell (Heidelberg, Germany). SPL Life Sciences (Pocheon, Republic of Korea) provided the adherent and ultra-low attachment (ULA) plates in 6- and 96-well sizes. Primers were acquired from Integrated DNA Technologies (Coralville, IA, USA) and TIB MOLBIOL (Berlin, Germany). Merck (St. Louis, MO, USA) and Panreac (Barcelona, Spain) provided the routine reagents.

### 2.2. Cell Culture and Mammosphere Generation

Breast cancer cells were maintained in DMEM supplemented with 10% FBS and 1% antibiotics at 37 °C with 5% CO_2_. T47D, MCF7, and MDA-MB-231 were seeded at a density of 2.5 × 10^5^ cells/well, 2 × 10^5^ cells/well, and 1.5 × 10^5^ cells/well, respectively, in adherent 6-well plates 24 h prior to treatment. Cells were treated with vehicle (0.01% DMSO) or ELIT cocktail (10 nM 17β-estradiol, 100 ng/mL leptin, 50 ng/mL interleukin-6, and 10 ng/mL TNFα) in phenol red-free DMEM (to avoid any estrogenic effect of phenol red) containing 10% FBS and 1% antibiotics for 48 h [9]. For mammosphere generation, single-cell suspensions of cells pre-exposed for 48 h to vehicle (CTRL) or ELIT cocktail were seeded in a ULA 6-well plate (4 × 10^4^ cells/well) or ULA 96-well plate (1 × 10^3^ cells/well) and cultured in 3DTM medium, containing CTRL or ELIT, to permit anchorage-independent growth in a Memmert^®^ ICO105 incubator (Schwabach, Germany) with 5% CO_2_ in a humidified atmosphere at 37 °C. After four days, the production of primary mammospheres was assessed using a 100× magnification inverted microscope.

### 2.3. Mammosphere Formation Efficiency and Size Determination

Following the generation of mammospheres, an inverted microscope set to 100× magnification was used to count all spheres with a diameter of ≥40 μm from each p96-well. ImageJ software was used to determine size and area. The following formula was utilized to determine the mammospheres formation efficiency (MFE): MFE (%) = (number of mammospheres generated per well)/(number of cells seeded per well) × 100, as previously described [25].

### 2.4. RNA Isolation and RT-qPCR

Following the manufacturer’s instructions, Tri Reagent^®^ Merck (St. Louis, MO, USA) was used to extract the total RNA from T47D, MCF7, and MDA-MB-231 mammospheres. A BioSpec-nano spectrophotometer (Shimadzu Biotech, Kyoto, Japan) was used to evaluate the concentration and purity of RNA. It was set to 260 nm and 280 nm, yielding a 260/280 nm ratio. Retrotranscription was used to generate cDNA, and PCR reactions were performed as previously reported [28]. Table S1 lists the genes with their primers and annealing temperatures. The Cp values of the qPCR were analyzed using the GenEx Standard Software (MultiD Analyses, Gothenburg, Sweden), normalizing with 18S as the housekeeping gene.

### 2.5. Cell Viability

Cell viability was determined using a fluorometric assay by staining DNA with Hoechst 33342 Merck (St. Louis, MO, USA), as previously described [29]. Mammospheres were dissociated and 1 × 10^4^ 3D-derived cells/well were seeded in adherent 96-well plates in phenol red-free DMEM containing 10% FBS and 1% antibiotics. After 24 h, cells were treated with vehicle (0.01% DMSO), set as CTRL; 10 µM Tamoxifen; or 10 nM Paclitaxel in the presence or absence of the ELIT cocktail, using 3DTM supplemented with Supplementation Mix for 48 h. DNA was stained with 0.01 mg/mL Hoechst 33342 in PBS, and the plate was incubated for 5 min at 37 °C in a 5% CO_2_ atmosphere. An FLx800 microplate fluorescence reader (BIO-TEK, Winooski, VT, USA) was used to measure fluorescence. The excitation and emission wavelengths were adjusted at 360 nm and 460 nm, respectively.

### 2.6. Measurement of H_2_O_2_ Production

Amplex^®^ Red Hydrogen Peroxide/Peroxidase Assay Kit (#A22188, Invitrogen, Waltham, MA, USA) was used to determine H_2_O_2_ production, as described before [30]. A total of 1 × 10^4^ 3D-derived cells/well were seeded in adherent 96-well plates in phenol red-free DMEM containing 10% FBS and 1% antibiotics. The next day, cells were treated with 10 µM Tamoxifen or 10 nM Paclitaxel in presence or not of the ELIT cocktail in 3DTM supplemented with Supplementation Mix for 48 h. Fluorescence was measured after cells were incubated with 0.1 U/mL HRP and 50 µM Amplex^®^ Red in Krebs–Ringer buffer. Hoechst 33342 was used to standardize the values to cell viability, as described before.

### 2.7. Data Collection

The GSE189757 dataset used in this study was obtained from the Gene Expression Omnibus (GEO; https://www.ncbi.nlm.nih.gov/geo, accessed on 16 July 2024) database. GSE189757 includes data from 44 women diagnosed with luminal breast cancer prior to any neoadjuvant treatment. Information regarding the inclusion and exclusion criteria of the original cohorts is available in the GSE189757 database. From all the samples of the identified dataset, data from 16 obese (Body Mass Index (BMI) ≥ 30) and 10 lean (BMI < 25) luminal breast cancer patients were used for Gene Set Enrichment Analysis (GSEA).

### 2.8. Gene Set Enrichment

Differentially Expressed Genes (DEGs) with |Fold Change (FC)| ≥ 1 and a *p*-value  ≤  0.05 were used to acquire Normalized Enrichment Scores (NES) of Gene Sets using GSEA software (number of permutations: 1000; excluding smaller sets of 15) [31,32]. Only gene sets with a *p*-value ≤ 0.05 and a False Discovery Rate (FDR) value ≤ 0.05 were considered significantly enriched.

### 2.9. ROC Analysis

The ROCplotter tool (www.rocplot.org, accessed on 18 July 2024) was used to analyze the expression levels of biomarkers previously identified through in vitro experiments. In a cohort of patients with luminal breast cancer, responders and non-responders to chemotherapy were compared. Pathological complete response (pCR) and relapse-free survival (RFS) at 5 years were assessed (N = 966 and N = 250, respectively). Additionally, analysis of pCR and RFS were performed, separating luminal A (N = 475 and N = 74, respectively) and luminal B (N = 491 and N = 173, respectively) subtypes as well as the HER2+ (N = 193 and N = 61, respectively) and TNBC (N = 473 and N = 164, respectively) molecular subtypes. To assess the predictive potential of the found genes, an ROC curve with a *p*-value ≤ 0.05 was determined to be significant between the two groups [33].

### 2.10. Statistical Analysis

The statistical analysis was conducted using the Statistical Program for the Social Sciences program for Windows (SPSS, version 27.0; SPSS Inc., Chicago, IL, USA). Five independent experiments (n = 5) were performed for 3D culture assays and three independent experiments (n = 3) were performed for 3D-derived cell assays. Data are presented as mean ± standard error of the mean (SEM). The statistical differences between CTRL cells and ELIT-exposed cells were assessed by Student’s *t*-test. Two-way analysis of variance (ANOVA) was performed to analyze the differences between these groups together with an interactive effect of Paclitaxel or Tamoxifen. The DMS test was then used as a post hoc comparison. Statistical significance was set as a *p*-value ≤ 0.05.

## 3. Results

### 3.1. Aggressiveness in Luminal Breast Cancer Is Promoted by Obesity-Related Inflammation

**Figure 1:**
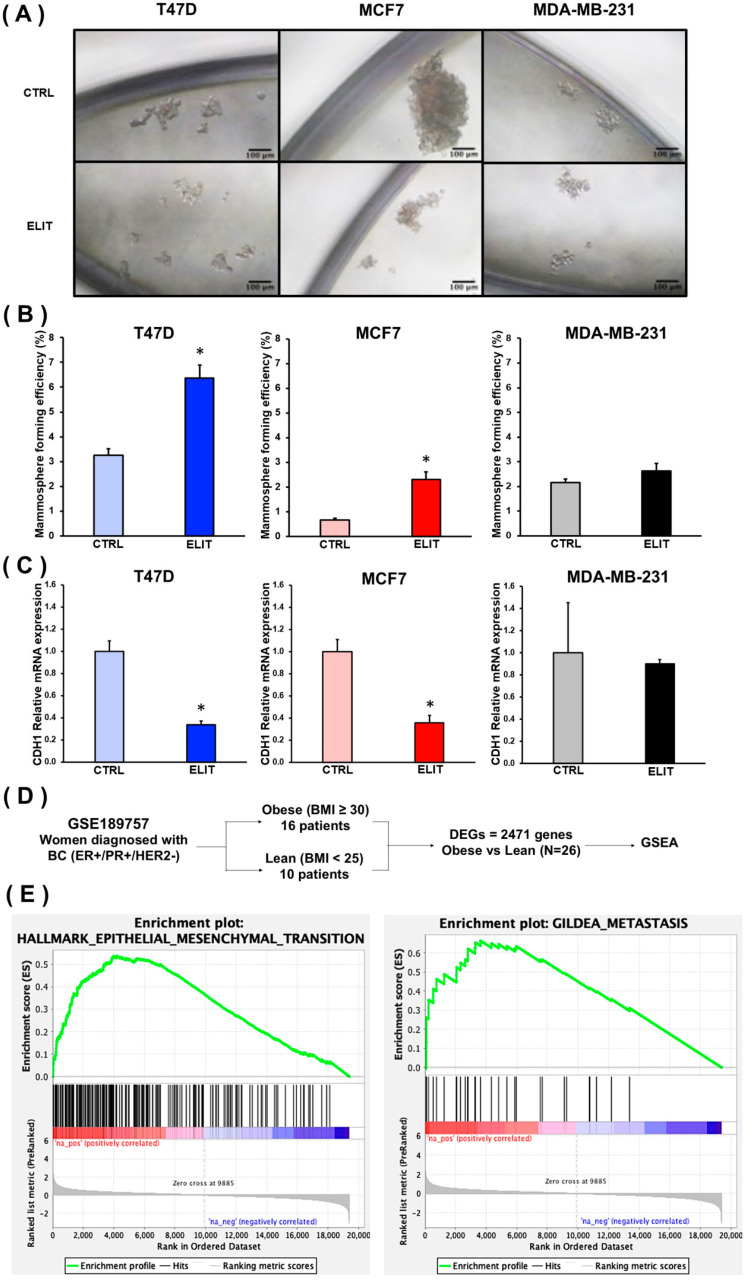
**Obesity-related inflammation induces aggressiveness in luminal breast cancer.** Mammospheres of T47D, MCF7, and MDA-MB-231 cell lines obtained after exposure to vehicle (CTRL) or ELIT cocktail (**A**). Mammosphere forming efficiency (MFE) of T47D, MCF7, and MDA-MB-231 cell lines under CTRL or ELIT conditions (**B**). mRNA expression levels of *CDH1* in mammospheres in CTRL or ELIT condition (**C**). Data are presented as mean ± SEM. Statistical significance was analyzed by Student’s *t*-test and set at * *p* ≤ 0.05. Enrichment analysis of Gene Sets related to aggressiveness in obese vs. lean luminal breast cancer patients from GSE189757 database (**D**,**E**). BMI: Body Mass Index; GSEA: Gene Set Enrichment Analysis.

Given that 3D culture systems (Figure 1A) closely mimic the physiological environment, mammosphere forming efficiency (MFE) was evaluated in luminal (T47D and MCF7) and triple-negative (MDA-MB-231) breast cancer cell lines exposed to the ELIT cocktail. The diameter of the spheres was also measured. As shown in Figure 1B, no significant difference in MFE was observed in MDA-MB-231 cells exposed to ELIT; however, T47D and MCF7 cells exhibited a marked increase in MFE. In addition, the diameter of mammospheres decreased in T47D and MCF7 breast cancer cell lines in the ELIT condition (Figure S1). Furthermore, mRNA expression levels of *CDH1*, a gene related to epithelial-to-mesenchymal transition (EMT) and metastasis pathways, were significantly higher in ELIT-exposed mammospheres of T47D and MCF7 cell lines (*p* = 0.001 and *p* = 0.002, respectively) compared to the control condition (Figure 1C).

To explore pathways associated with the aggressiveness of obesity-related conditions in luminal breast cancer, GSEA software was used to analyze pathway enrichment of Gene Sets across all DEGs (|Fold Change (FC)| ≥ 1 and a *p*-value ≤ 0.05) between obese and lean luminal breast cancer patients (Figure 1D). As illustrated in Figure 1E, breast cancer patients with obesity showed a positive NES in the pathways *Epithelial–Mesenchymal Transition* and *Metastasis*.

### 3.2. ELIT Exposure Decreases Oxidative Stress and Mitochondrial Markers in Luminal Breast Cancer Mammospheres and Increases ESR2 mRNA Expression

**Figure 2:**
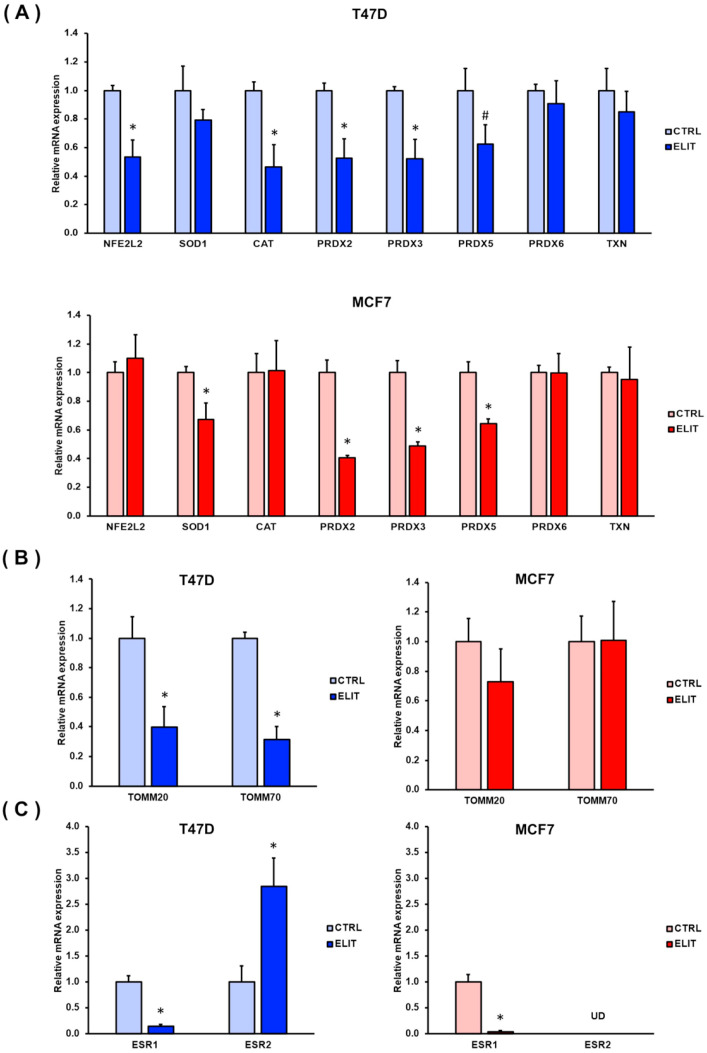
**ELIT exposure modifies antioxidant, mitochondrial markers in luminal breast cancer mammospheres with high *ESR2* mRNA expression.** mRNA expression levels of oxidative stress-related genes (**A**), mitochondrial markers (**B**), and *ESR1* and *ESR2* (**C**) in T47D and MCF7 mammospheres under CTRL or ELIT condition. Data are presented as mean ± SEM. Statistical significance was analyzed by Student’s *t*-test and set at * *p* ≤ 0.05 and # *p* ≤ 0.1. UD: Undetected values.

To further investigate the oxidative stress response and mitochondrial status of luminal breast cancer cell-derived mammospheres following ELIT exposure, the mRNA expression levels of antioxidant-related genes and mitochondrial markers were analyzed. As shown in Figure 2A, T47D mammospheres exhibited a statistically significant decrease in the expression of antioxidant markers, including *NFE2L2* (*p* = 0.006), *CAT* (*p* = 0.022), *PRDX2* (*p* = 0.011), *PRDX3* (*p* = 0.027), and *PRDX5* (*p* = 0.1). On the other hand, MCF7 mammospheres demonstrated a significant reduction in *SOD1* (*p* = 0.014), *PRDX2* (*p* = 0.001), *PRDX3* (*p* = 0.001), and *PRDX5* (*p* = 0.006) mRNA expression. MDA-MB-231 cell line’s mRNA expression data are displayed in Figure S3.

To assess the effect of ELIT condition on mitochondrial status, considering that mitochondria are the main source of ROS production, the mRNA expression of translocases located in the outer mitochondrial membrane (TOMM) was analyzed. As shown in Figure 2B, no significant changes were observed in MCF7 mammospheres. Nevertheless, T47D mammospheres displayed a notable decrease in *TOMM20* (*p* = 0.021) and *TOMM70* (*p* < 0.001) mRNA expression levels after ELIT exposure. The MDA-MB-231 cell line showed no statistically significant differences (Figure S4).

Additionally, the mRNA expression of both estrogen receptor alpha (*ESR1*) and beta (*ESR2*) was analyzed to further understand the differential response of luminal breast cancer cell lines with distinct basal levels of *ESR2* expression (Figure 2C). Both T47D and MCF7 mammospheres exhibited a nearly complete loss of *ESR1* mRNA expression (*p* = 0.002 and *p* = 0.001, respectively). Interestingly, while *ESR2* mRNA levels were undetectable in MCF7 mammospheres, a significant increase in *ESR2* expression was observed in T47D mammospheres in ELIT condition (*p* = 0.043).

### 3.3. ELIT Condition Impairs Drug Response in T47D 3D-Derived Cells

**Figure 3:**
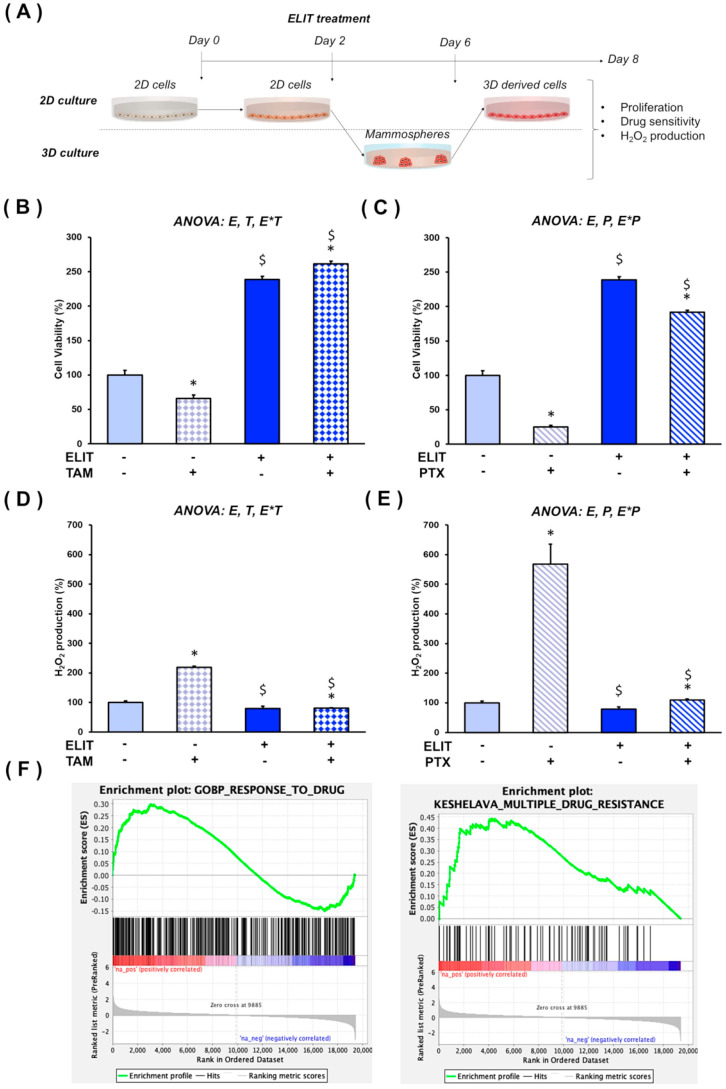
**ELIT exposure decreases drug sensitivity of T47D 3D-derived cells.** Experimental design for T47D 3D-derived cells analysis (**A**). Cell viability (**B**,**C**) and H_2_O_2_ production (**D**,**E**) in T47D 3D-derived cells under CTRL or ELIT condition after treatment with Tamoxifen or Paclitaxel, respectively. Enrichment analysis of Gene Sets related to drug response in obese compared to lean luminal breast cancer patients from GSE189757 database (**F**). ANOVA analysis was carried out, where E means ELIT effect; T means tamoxifen effect; P means Paclitaxel effect; and E*T or E*P mean interactive effect of ELIT with Tamoxifen or Paclitaxel, respectively. Data are presented as mean ± SEM. * Significant difference between cells treated with Tamoxifen or Paclitaxel and untreated cells (*p* ≤ 0.05). $ Significant differences between CTRL and ELIT-exposed cells (*p* ≤ 0.05).

To evaluate the effect of ELIT on cell viability after 3D culture, which may simulate the colonization process of breast cancer cells, 3D-derived cells from T47D and MCF7 mammospheres were analyzed (Figure 3A). MCF7 3D-derived cells exposed to ELIT exhibited a significant reduction in cell viability, whereas T47D 3D-derived cells displayed a statistically significant increase in viability (see Supplementary Data Table S2).

Furthermore, both cell viability and H_2_O_2_ production were measured after ELIT exposure to assess the response of T47D 3D-derived cells to Tamoxifen and Paclitaxel, two drugs commonly used to treat luminal breast cancer. In contrast to CTRL cells, ELIT exposure increased cell viability after Tamoxifen treatment (Figure 3B). On the other hand, ELIT-exposed cells were less sensitive to Paclitaxel compared to the CTRL condition (Figure 3C). Additionally, Figure 3D,E show that ELIT exposure significantly reduced ROS production in T47D cells in response to Tamoxifen and Paclitaxel when compared to the CTRL condition.

Finally, we assessed the clinical relevance of our findings in luminal breast cancer patients with obesity. Enrichment analysis revealed a positive NES in the pathways *Response to drug* and *Multiple drug resistance* (Figure 3F).

### 3.4. Identification of Biomarkers Related to Poor Prognosis in Patients with Luminal Breast Cancer

The ROCplotter bioinformatic tool was used to evaluate the relevance of specific differentially expressed genes in T47D mammospheres after ELIT exposure as potential biomarkers of poor prognosis (pCR and RFS) in responders and non-responders to chemotherapy of luminal breast cancer patients.

**Figure 4:**
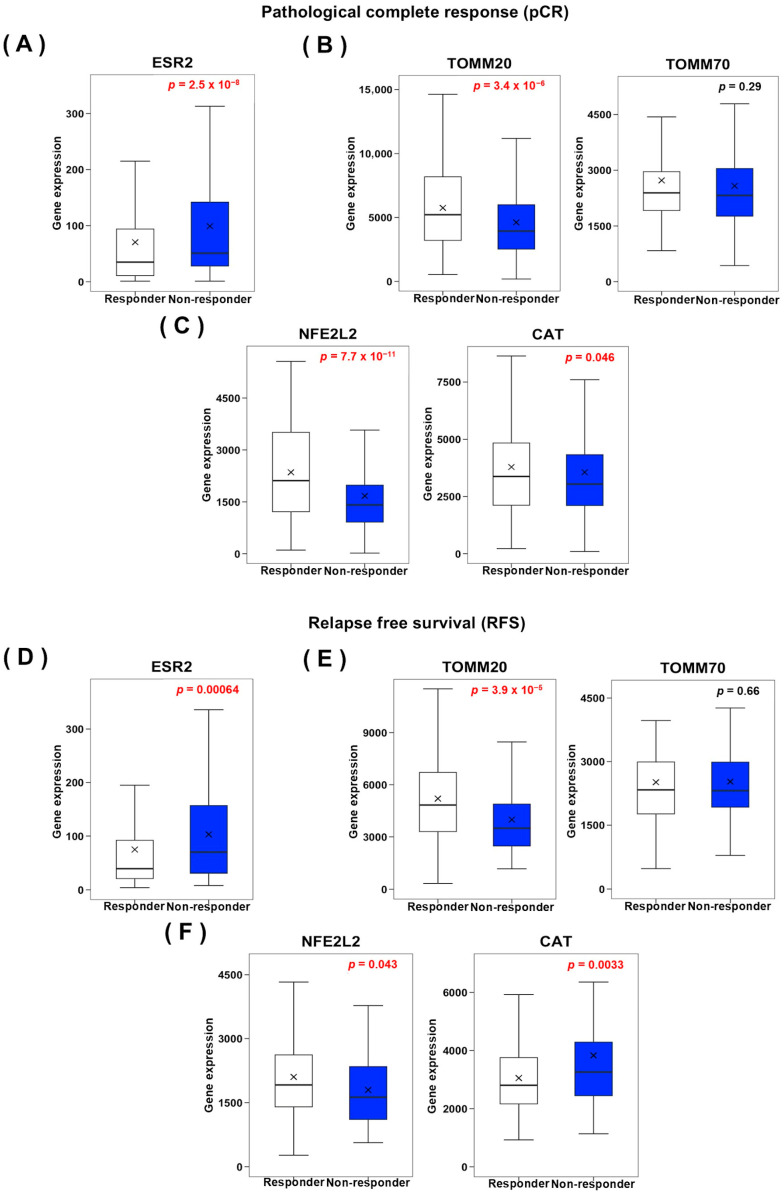
**Obesity-related biomarkers associated with poor prognosis in luminal breast cancer.** Gene expression of *ESR2* (**A**) and mitochondrial (**B**) and oxidative stress (**C**) markers in luminal breast cancer responder and non-responder patients according to pCR after chemotherapy. Gene expression of *ESR2* (**D**) and mitochondrial (**E**) and oxidative stress (**F**) markers in luminal breast cancer responder and non-responder patients according to RFS after chemotherapy. Statistical significance was analyzed by Student’s *t*-test and set at *p* ≤ 0.05 (highlighted values).

As illustrated in Figure 4A, *ESR2* mRNA levels (*p* = 2.5 × 10^−8^) were significantly higher in breast cancer patients who did not achieve a pCR following chemotherapy. Conversely, *TOMM20* (*p* = 3.4 × 10^−6^), *NFE2L2* (*p* = 7.7 × 10^−11^), and *CAT* (*p* = 0.046) mRNA levels were significantly lower in patients who did not respond to treatment (Figure 4B,C).

Furthermore, *ESR2* (*p* = 0.00064) and *CAT* (*p* = 0.0033) mRNA levels were elevated in patients who did not experience RFS after chemotherapy, while *TOMM20* (*p* = 3.9 × 10^−5^) and *NFE2L2* (*p* = 0.043) mRNA levels were reduced in non-responders (Figure 4D,F).

Complementarily, the impact of these biomarkers on pCR (Figure S4) and RFS (Figure S5) was also analyzed, separating luminal A and luminal B subtypes as well as the HER2+ and TNBC molecular subtypes.

## 4. Discussion

This study explores the interplay between obesity-related inflammation, as simulated by the ELIT cocktail, and breast cancer progression. Our results reveal how key inflammatory factors can affect mammosphere formation, antioxidant and mitochondrial markers, and response to standard therapies [7]. These findings highlight the critical role of obesity-related inflammation in enhancing the aggressiveness of luminal breast cancer, suggesting that this environment may influence the expression of *ESR2* as well as oxidative stress and mitochondrial biomarkers.

Over the past few decades, obesity has been recognized as a promoter of tumor migration by inducing an inflammatory environment that could stimulate the spread of cancer cells [34,35]. In the present study, we exposed breast cancer cells to the ELIT cocktail (17β-estradiol, leptin, IL6, and TNFα) simulating the hormonal and inflammatory conditions of postmenopausal obesity [9]. Our research group has previously demonstrated that the mammosphere model employed in the present study is a valuable approach for analyzing biomarkers related to tumor aggressiveness in breast cancer [25]. Here, we found an increased mammosphere forming efficiency in luminal breast cancer cells following ELIT exposure, suggesting that obesity-related inflammation enhances the aggressive features of these cancer cells. The GSEA analysis conducted in luminal breast cancer patients with obesity revealed altered cellular signaling pathways, indicating an increased ability for cancer cells to invade surrounding tissues and promote metastases [36]. Accordingly, the reduction in *CDH1* mRNA expression in ELIT-exposed luminal mammospheres further supports the involvement of EMT, a process that facilitates cancer cell dissemination and invasion [37,38]. Together, these observations support the idea that luminal breast cancers, with high reliance on estrogen signaling, may be particularly vulnerable to the influence of obesity [7,36]. In fact, these effects were not found in the triple-negative MDA-MB-231 cell line, which lacks *ESR1* expression, again suggesting a specific dependency on estrogen signaling in mediating the effects of obesity-related inflammation.

Indeed, epidemiological studies also support this connection, as obese patients with luminal tumors tend to have poorer clinical outcomes, likely due to the complex interplay between estrogen signaling and inflammatory pathways [36,39,40]. Despite the elevated circulating estrogen levels commonly observed in obese individuals [6,11], the strong reduction in *ESR1* expression observed in luminal breast cancer mammospheres after ELIT exposure is consistent with a previous study in adherent cells [9]. This reduction in *ESR1* has also been reported with 17β-estradiol in rat uterus and several breast cancer cell lines, including T47D and MCF7 [41,42,43]. Interestingly, the increased expression of *ESR2* under ELIT condition found in the T47D cell line, which expresses the highest baseline levels of *ESR2*, highlights the potential role of *ESR2* in modulating cellular responses under inflammatory conditions [9]. These interactions underscore the complex relationship between inflammation and hormonal regulation, particularly in luminal breast cancer patients [39].

Several studies have reported that *ESR2* is involved in the regulation of mitochondrial function, oxidative stress, and cellular energy metabolism in cancer [43,44,45]. Our results support these findings, specifically in the T47D cell line, with an increase in *ESR2* and a decrease in mitochondrial markers *TOMM20* and *TOMM70*, as well as in antioxidant-related markers *NFE2L2* and *CAT*, which are critical for maintaining cellular redox balance [21,46]. Most of these markers altered by obesity-related inflammation were also correlated with a poorer prognosis in luminal breast cancer patients in our in silico analysis, being particularly relevant in pCR for the luminal A subtype and in RFS for the luminal B subtype. Similarly, Kolb et al. found a marker increased by obesity-associated inflammation, ANGPTL4, which was also correlated with a poor prognosis of breast cancer patients [47]. Notably, *TOMM20* and *TOMM70*, key components of the TOMM complex, play a crucial role in the mitochondrial import system [48,49]. The TOMM complex is essential for maintaining mitochondrial integrity and function, which reflects the overall status of the mitochondria [48,50]. Since mitochondria are the main source of ROS, we also investigated key antioxidant regulators. *NFE2L2*, the master regulator of antioxidant response, is closely associated with cancer cell survival, drug resistance, and metastasis [21,51,52]. Although its role in breast cancer progression remains controversial, some studies suggest that high *NFE2L2* levels enhance antioxidant responses, promoting chemoresistance and metastasis [21,52]. Conversely, lower *NFE2L2* levels have been associated with poorer prognosis, particularly in luminal breast cancer, which is consistent with our findings [53]. In fact, our data suggest that reduced *NFE2L2* mRNA levels correlate with a worse response to treatment in terms of pCR and RFS in luminal breast cancer. Similarly, *CAT*, another key enzyme involved in the detoxification of ROS, also showed altered expression in cancer [54]. While higher catalase activity has been associated with a less aggressive cancer phenotype and reduced metastatic potential [55], its reduction here suggests a weakened antioxidant defense.

Recent research has shown that disruptions in redox balance, oxidative stress, and mitochondrial function may be key factors in determining tumor aggressiveness and resistance to therapies [23,46,56,57]. Spreader cancer cells acquire an invasive phenotype largely driven by EMT, which enables them to migrate and disseminate to distant organs [58,59]. The re-culturing of 3D-derived luminal breast cancer cells under adherent conditions allowed us to mimic, in part, the behavior of disseminated cancer cells. The reduced sensitivity to common therapeutic drugs—Tamoxifen and Paclitaxel [60,61]—observed in T47D cells exposed to an obesity-related inflammatory condition, together with a decrease in ROS production, supports that chronic inflammation, particularly in the context of obesity, may condition luminal breast cancer cells by disrupting their redox balance and enhancing their ability to resist the effects of treatments [23,46]. Thus, our findings suggest that obesity-related inflammation compromises the efficacy of standard treatments that increase ROS production and reduce cell viability [8,62] likely through mechanisms involving oxidative stress and mitochondrial dysfunction [7,63,64]. Notably, recent studies have reported a correlation between high BMI and increased resistance to therapies in luminal breast cancer, which is linked to the inflammatory microenvironment characteristic of obesity [65,66]. In particular, components such as IL6 and leptin have been demonstrated to play a significant role in resistance to treatments like Tamoxifen and Paclitaxel in breast cancer patients [67,68]. Consistently, our GSEA analysis revealed the upregulation of *Response to drug* and *Multiple drug resistance* pathways in obese luminal breast cancer patients compared to their lean counterparts, further highlighting the potential impact of obesity in modulating treatment response in these patients.

Taken together, the increased expression of *ESR2*, combined with the reduced expression of key mitochondrial and antioxidant regulators, suggests that obesity-related inflammation enhances the aggressive phenotype of luminal breast cancer through a compromised mitochondrial and antioxidant response.

## 5. Conclusions

In conclusion, our findings indicate that markers including *ESR2*, *TOMM20*, *NFE2L2*, and *CAT* not only play a role in mitochondrial oxidative stress but may also influence drug response and cancer recurrence, making them promising biomarkers to predict prognosis in luminal breast cancer. Thus, this study provides a potential understanding for future research into targeted therapies designed to mitigate the negative effects of obesity on luminal breast cancer outcomes, with a specific emphasis on mitochondrial function and oxidative stress pathways.

## Data Availability

The data presented in this study are available on request from the corresponding author.

## Supplementary Materials

The following supporting information can be downloaded at: https://www.mdpi.com/article/10.3390/biomedicines12122813/s1, Figure S1: Sphere number of T47D, MCF7, and MDA-MB-231 mammospheres after ELIT exposure according to diameter (µm); Figure S2: mRNA expression levels of oxidative stress-related genes in MDA-MB-231 mammospheres under CTRL or ELIT condition; Figure S3: mRNA expression levels of mitochondrial markers in MDA-MB-231 mammospheres under CTRL or ELIT condition; Figure S4: Gene expression of *ESR2*, *TOMM20*, *TOMM70*, *NFE2L2*, and *CAT* in luminal A, luminal B, HER2+ and TNBC breast cancer responder and non-responder patients according to pCR after chemotherapy; Figure S5: Gene expression of *ESR2*, *TOMM20*, *TOMM70*, *NFE2L2*, and *CAT* in luminal A, luminal B, HER2+ and TNBC breast cancer responder and non-responder patients according to RFS after chemotherapy; Table S1: Primer sequences and annealing temperature for mRNA expression analysis using qPCR; Table S2: Cell viability of T47D and MCF7 3D-derived cells after ELIT exposure.
