# Supplementary material for: Obesity-Related Inflammation Reduces Treatment Sensitivity and Promotes Aggressiveness in Luminal Breast Cancer Modulating Oxidative Stress and Mitochondria
Source: Biomedicines. 2024 Dec 11;12(12):2813. doi: 10.3390/biomedicines12122813 (PMC11673959; doi:10.3390/biomedicines12122813)
Supplement: Supplementary file 1 [file biomedicines-12-02813-s001.zip › biomedicines-3317677-supplementary.pdf]

## Supplementary data

**Table S1.** Primer sequences and annealing temperature for mRNA expression analysis using qPCR.

| Gene | Forward Primer (5'–3')<br>Reverse Primer (5'–3') | Annealing Temperature (°C) |
|---|---|---|
| *18S* | GGACACGGACAGGATTGACA<br>ACCCACGGAATCGAGAAAGA | *60* |
| *CDH1* | GTCACTGACACCAACGATAATCCT<br>TTTCAGTGTGGTGATTACGACGTTA | *61* |
| *NFE2L2* | GCG ACG GAA AGA AGT ATG AGC<br>GTT GGC AGA TCC ACT GGT TT | *60* |
| *SOD1* | TCA GGA GAC CAT TGC ATC ATT<br>CGC TTT CCT GTC TTT GTA CTT TCT TC | *64* |
| *CAT* | CAT CGC CAC ATG AAT GGA TA<br>CCA ACT GGG ATG AGA GGG TA | *61* |
| *PRDX2* | CCA GAC GCT TGT CTG AGG AT<br>ACG TTG GGC TTA ATC GTG TC | *60* |
| *PRDX3* | GCC GTT GTC AAT GGA GAG TT<br>TCC ACT GAG ACT GCG ACA AC | *60* |
| *PRDX5* | ACG GTG CAG TGA AGG AGA GT<br>CAG GAA CTC CAA ACA GCA CA | *60* |
| *PRDX6* | CGT GTG GTG TTT GTT TTT GG<br>CCA TCA CAC TAT CCC CAT CC | *60* |
| *TXN* | CTG CTT TTC AGG AAG CCT TG<br>TGT TGG CAT GCA TTT GAG TT | *60* |
| *TOMM20* | ACTGTGTTTGTGAAAACTGAGCA<br>CTAGCGAAGCTCACAAGGCT | *58* |
| *TOMM70* | GGGTTGTGTTCTTCAGGGGT<br>CAGCGGTCTCAAACTTCCCA | *60* |
| *ESR1* | AAT TCA GAT AAT CGA CGC CAG<br>GTG TTT CAA CAT TCT CCC TCC TG | *61* |
| *ESR2* | TAG TGG TCC ATC GCC AGT TAT<br>GGG AGC CAC ACT TCA CCA T | *58* |

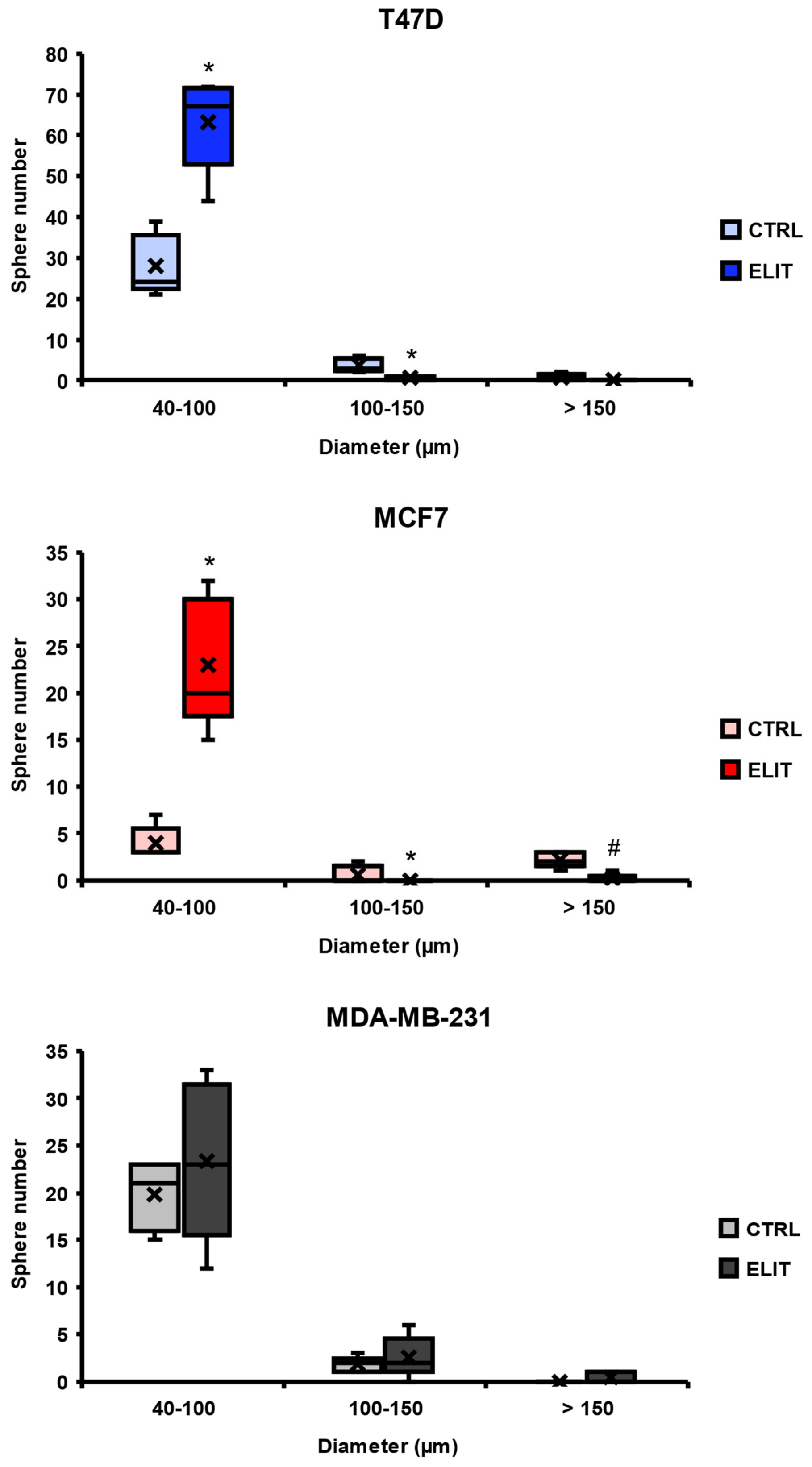


**Figure S1.** Sphere number of T47D, MCF7, and MDA-MB-231 mammospheres after ELIT exposure according to diameter (µm). Data are presented as mean ± SEM. Statistical significance of differences between ELIT-treated and CTRL cells was analyzed by Student's t-test and set at * $p \leq 0.05$ and # $p \leq 0.1$.

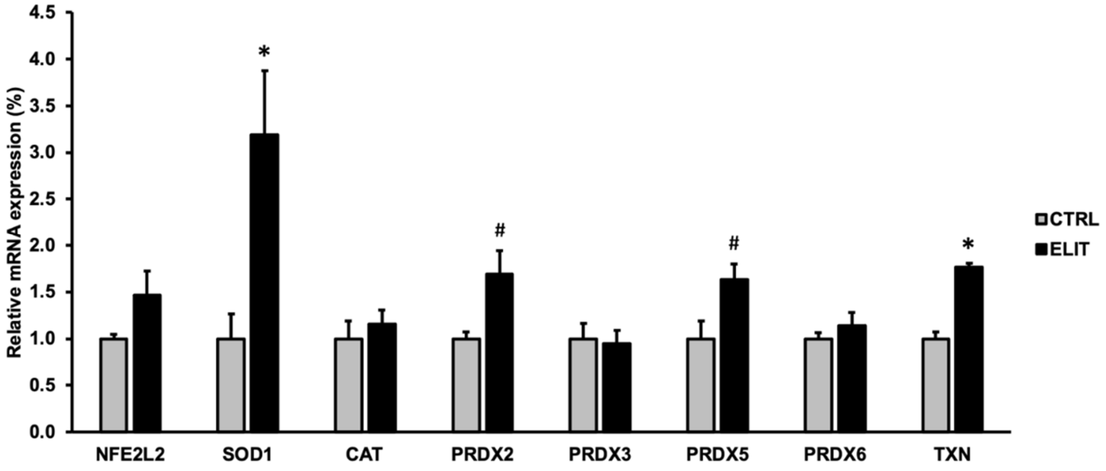


**Figure S2.** mRNA expression levels of oxidative stress-related genes in MDA-MB-231 mammospheres under CTRL or ELIT condition. Data are presented as mean ± SEM. Statistical significance was analyzed by Student's t-test and set at * $p \leq 0.05$ and # $p \leq 0.1$.

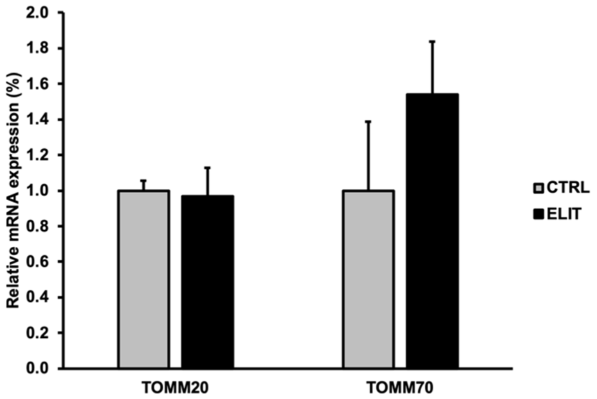


**Figure S3.** mRNA expression levels of mitochondrial markers in MDA-MB-231 mammospheres under CTRL or ELIT condition. Data are presented as mean ± SEM. Statistical significance was analyzed by Student's t-test.

**Table S2.** Cell viability of T47D and MCF7 3D-derived cells after ELIT exposure.

| | **CTRL** | **ELIT** |
|---|---|---|
| **T47D** | 100 ± 7 | 238 ± 5* |
| **MCF7** | 100 ± 2 | 15.5 ± 5.1* |

Data are presented as means ± SEM. Values of CTRL cells were set at 100. * Significant difference between ELIT-treated and control cells (Student's test; $p < 0.05$).

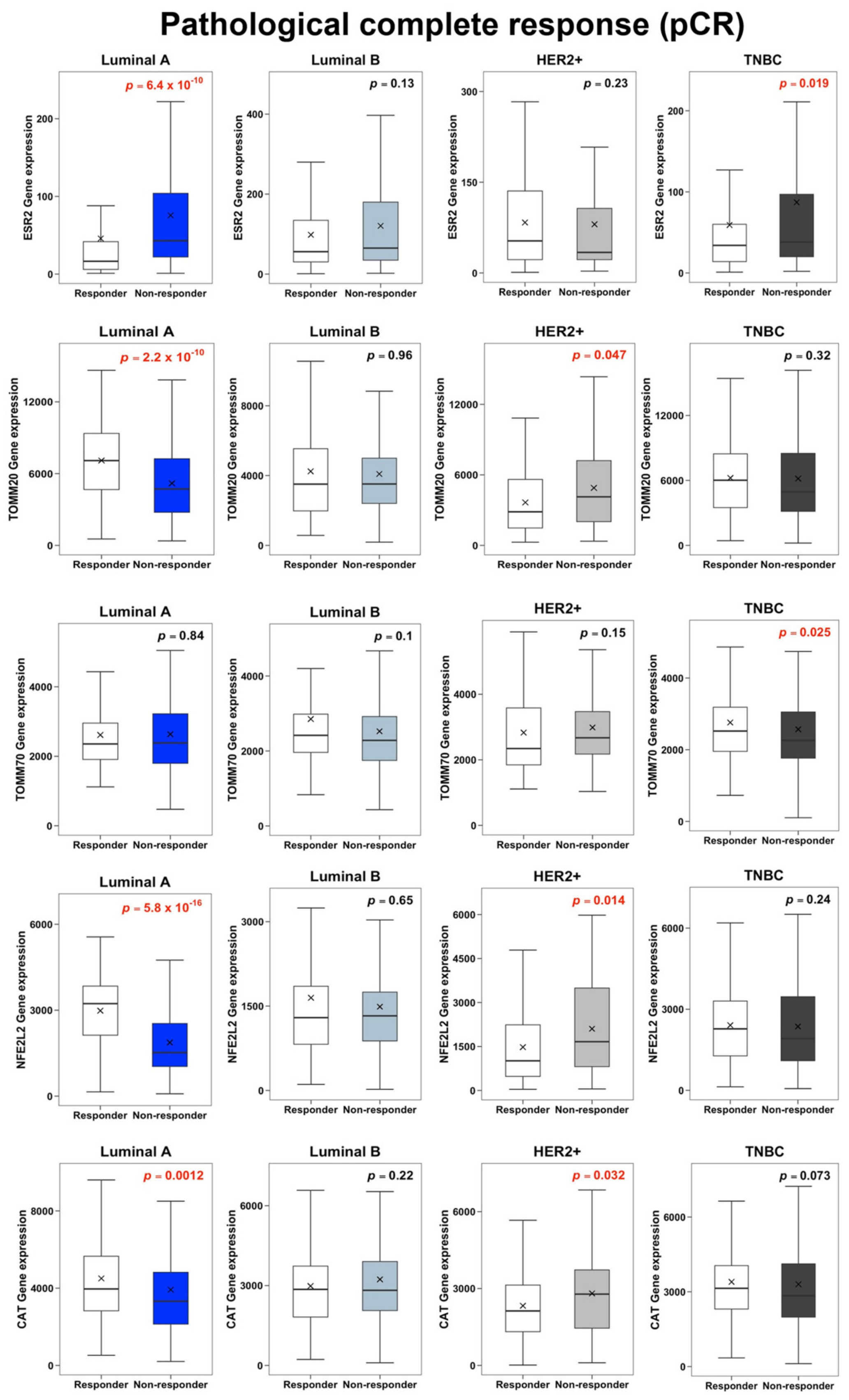


**Figure S4.** Gene expression of *ESR2, TOMM20, TOMM70, NFE2L2,* and *CAT* in luminal A, luminal B, HER2+ and TNBC breast cancer responder and non-responder patients according to pCR after chemotherapy. Statistical significance was analyzed by Student's t-test and set at p ≤ 0.05 (highlighted values).

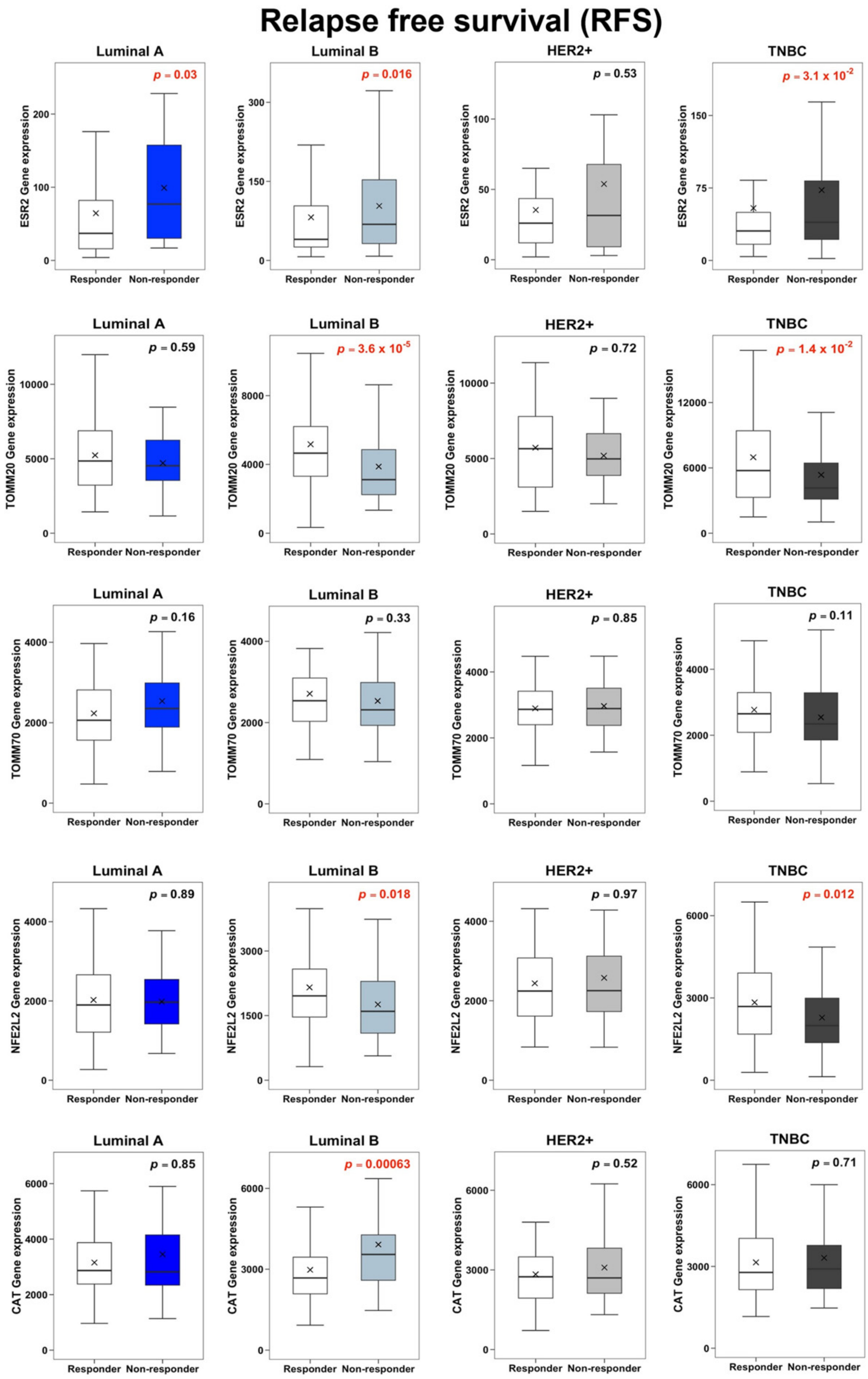


**Figure S5.** Gene expression of *ESR2, TOMM20, TOMM70, NFE2L2,* and *CAT* in luminal A, luminal B, HER2+ and TNBC breast cancer responder and non-responder patients according to RFS after chemotherapy. Statistical significance was analyzed by Student's t-test and set at $p \leq 0.05$ (highlighted values).
